# Crystal structure of a solvated dinuclear Cu^II^ complex derived from 3,3,3′,3′-tetraethyl-1,1′-(furan-2,5-dicarbonyl)bis(thiourea)

**DOI:** 10.1107/S2056989024010703

**Published:** 2024-11-08

**Authors:** Canh Dinh Le, Hoang Phuc Nguyen, Chien Thang Pham

**Affiliations:** a Department of Chemistry, Quy Nhon University, 170 An Duong Vuong, Quy Nhon, Vietnam; b Department of Inorganic Chemistry, VNU University of Science, Vietnam National, University, Hanoi, 19 Le Thanh Tong, Hanoi, Vietnam; Universidad de la República, Uruguay

**Keywords:** crystal structure, aroylbis(*N*,*N*-di­alkyl­thio­ureas), Cu(II) complexes

## Abstract

Reaction between equimolar amounts of 3,3,3′,3′-tetraethyl-1,1′-(furan-2,5-dicarbonyl)bis(thiourea) (**H_2_L**) and CuCl_2_·2H_2_O in methanol in the presence of the supporting base Et_3_N gave rise to a neutral dinuclear complex bis­[μ-3,3,3′,3′-tetraethyl-1,1′-(furan-2,5-dicarbonyl)bis(thioureato)]dicopper(II) di­chloro­methane disolvate, [Cu_2_(C_16_H_22_N_4_O_3_S_2_)_2_]·2CH_2_Cl_2_ or [Cu_2_(*L*)_2_]·2CH_2_Cl_2_. The aroylbis(thio­ureas) are doubly deprotonated and the resulting anions {*L*^2–^} bond to metal ions through (*S*,*O*)-chelating moieties. The copper atoms adopt a virtually *cis*-square-planar environment. In the crystal, adjacent [Cu_2_(*L*)_2_]·2CH_2_Cl_2_ units are linked into polymeric chains along the *a-*axis direction by inter­molecular coordinative Cu⋯S inter­actions. The co-crystallized solvent mol­ecules play a vital role in the crystal packing. In particular, weak C—H_furan_⋯Cl and C—H_eth­yl_⋯Cl contacts consolidate the three-dimensional supra­mol­ecular architecture.

## Abstract 2

In the title compound, [Cu_2_(*L*)_2_]·2CH_2_Cl_2_, the Cu^II^ ions coordinate two (*S*,*O*)-chelating aroyl­thio­urea moieties of doubly deprotonated furan-2,5-di­carbonyl­bis­(*N*,*N*-di­ethyl­thio­urea) (**H_2_L**) ligands. The coordination geometry of the metal centers is best described as a flat isosceles trapezoid with a *cis* arrangement of the donor atoms.

## 1. Chemical context

Benzo­yl(*N*,*N*-di­alkyl­thio­ureas) are versatile ligands forming stable complexes with a great number of transition-metal ions, in which the organic compounds mainly act as monoanionic and (*S*,*O*)-bidentate ligands (Fitzl *et al.*, 1977 ▸; Knuuttila *et al.*, 1982 ▸; Sieler *et al.*, 1990 ▸; Bensch *et al.*, 1995 ▸; Nguyen *et al.*, 2007 ▸; Barnard *et al.*, 2019 ▸; Pham *et al.*, 2021 ▸). This coordination fashion also plays an important role in metal complexes of aroylbis(thio­ureas), such as homo-dinuclear complexes based on the bipodal *iso*-phthaloylbis(*N*,*N*-di­alkyl­thio­ureas) (Koch *et al.*, 2001 ▸; Rodenstein *et al.*, 2008 ▸; Schwade *et al.*, 2013 ▸; Schwade *et al.*, 2020 ▸; Teixeira *et al.*, 2020 ▸). The presence of potential donor atom(s) in the spacer between two aroyl­thio­urea moieties, such as pyridine N or catechol O atoms, could enable the corresponding aroylbis(thio­ureas) to serve as building blocks for the construction of heteronuclear host–guest systems (Nguyen *et al.*, 2016 ▸; Pham *et al.*, 2017 ▸, 2020 ▸; Le *et al.*, 2019 ▸; Jesudas *et al.*, 2020 ▸). However, it seems such aroylbis(thio­ureas) are not appropriate for the production of homonuclear systems. Indeed, all efforts to produce related homonuclear complexes, as in the case of *iso*-phthaloylbis(*N*,*N*-di­alkyl­thio­ureas), have hitherto failed. Herein, we describe the synthesis and crystal structure of the first homonuclear complex derived from the novel 3,3,3′,3′-tetraethyl-1,1′-(furan-2,5-dicarbonyl)bis(thiourea) (**H_2_L**), referred to as furan-2,5-di­carbonyl­bis­(*N*,*N*-di­ethyl­thio­urea), which possesses a potential furan O donor atom in the mol­ecular backbone. The compound, [Cu_2_(*L*)_2_], potentially exhibits inter­esting magnetic and catalytic properties (Pham *et al.*, 2019 ▸; Nath *et al.*, 2020 ▸).
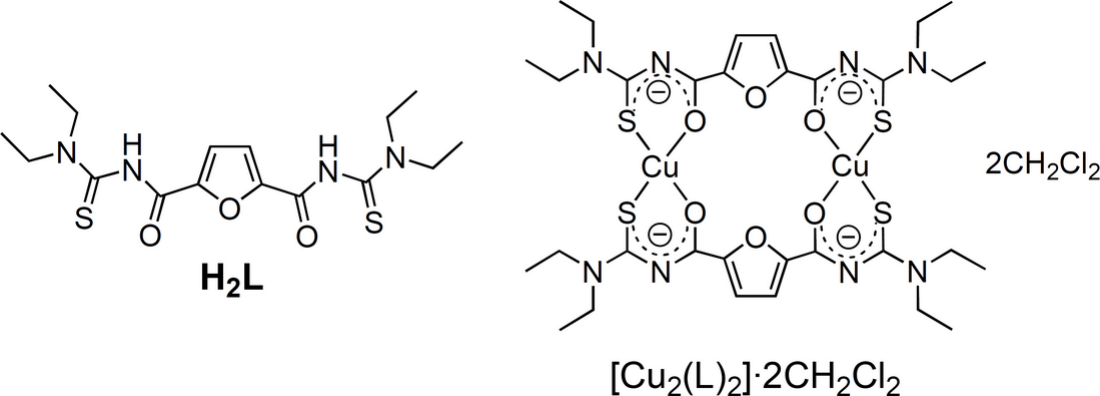


## 2. Structural commentary

**Figure 1:**
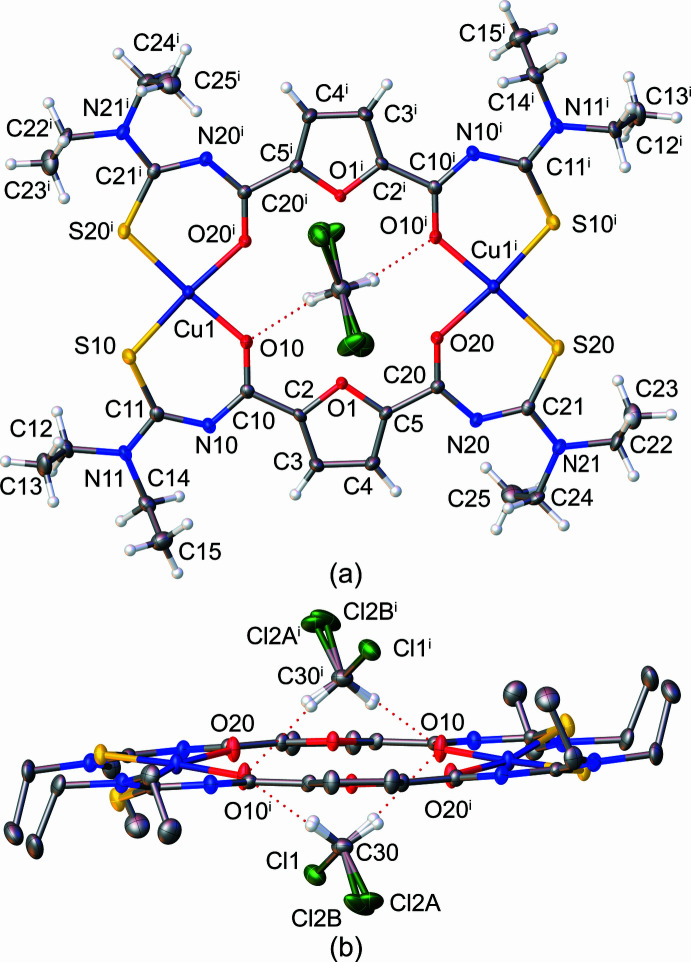
The mol­ecular structure of the title compound [Cu_2_(*L*)_2_]·2CH_2_Cl_2_. (*a*) Top view with complete labeling of non-hydrogen atoms within the complex mol­ecule. (*b*) Side view. Hydrogen atoms of the complex are omitted for clarity. Displacement ellipsoids are drawn at the 50% probability level. The red dotted lines indicate the C—H⋯O hydrogen bonds. Symmetry code: (i) −*x* + 1, −*y* + 1, −*z* + 1.

The complex [Cu_2_(*L*)_2_] crystallizes as a solvated form in the centrosymmetric monoclinic space group *P*2_1_/*n* with half of [Cu_2_(*L*)_2_]·2CH_2_Cl_2_ in the asymmetric unit. The mol­ecular structure, including solvent mol­ecules, is shown in Fig. 1 ▸. The complex consists of two Cu^II^ ions and two doubly deproton­ated ligands {*L*}^2–^, which bond to the metal ions through (*S*,*O*)-chelating aroyl­thio­urea moieties. The Cu1—O bond lengths are Cu1—O10 = 1.9406 (15) Å and Cu1—O20 = 1.9431 (14) Å, while the Cu1—S10 and Cu1—S20 bond lengths are 2.2624 (6) and 2.2612 (6) Å, respectively. These bond distances fall in the same ranges for those observed in several copper(II) complexes with aroylmono(thio­ureas) (Wu *et al.*, 2015 ▸; Selvakumaran *et al.*, 2016 ▸; Binzet *et al.*, 2018 ▸; Pham *et al.*, 2021 ▸) and aroylbis(thio­ureas) (Rodenstein *et al.*, 2008 ▸; Schwade *et al.*, 2013 ▸; Teixeira *et al.*, 2020 ▸). The metal⋯metal distance is 7.762 (3) Å and the midpoint between the two copper atoms is on the inversion center of the mol­ecule. The two chelate planes Cu1/O10–S10 (r.m.s.d. = 0.075 Å) and Cu1/O20^i^–S10^i^ (r.m.s.d. = 0.156 Å) form a dihedral angle of 15.32 (2)°. Thus, the four-coordinate Cu^II^ atoms adopt a flat isosceles trapezoid geometry due to the *cis* arrangement of the donor atoms. The atoms within the furan-2,5-dicarboxamide moieties and the copper atoms are nearly coplanar with a largest deviation of 0.298 (2) Å from the mean least-squares plane for the furan oxygen atoms. Two CH_2_Cl_2_ mol­ecules are located on either side of the plane at a distance of 1.991 (5) Å from the plane to the solvent carbon atoms. One chlorine atom of the solvent mol­ecule is disordered over two positions with occupancy factor of 0.6163 (9) for the atom labelled *A*. In addition, the solvent inter­acts with the complex through hydrogen bonds formed with the carbonyl oxygen atoms O10 (Table 1 ▸).

## 3. Supra­molecular features

**Figure 2:**
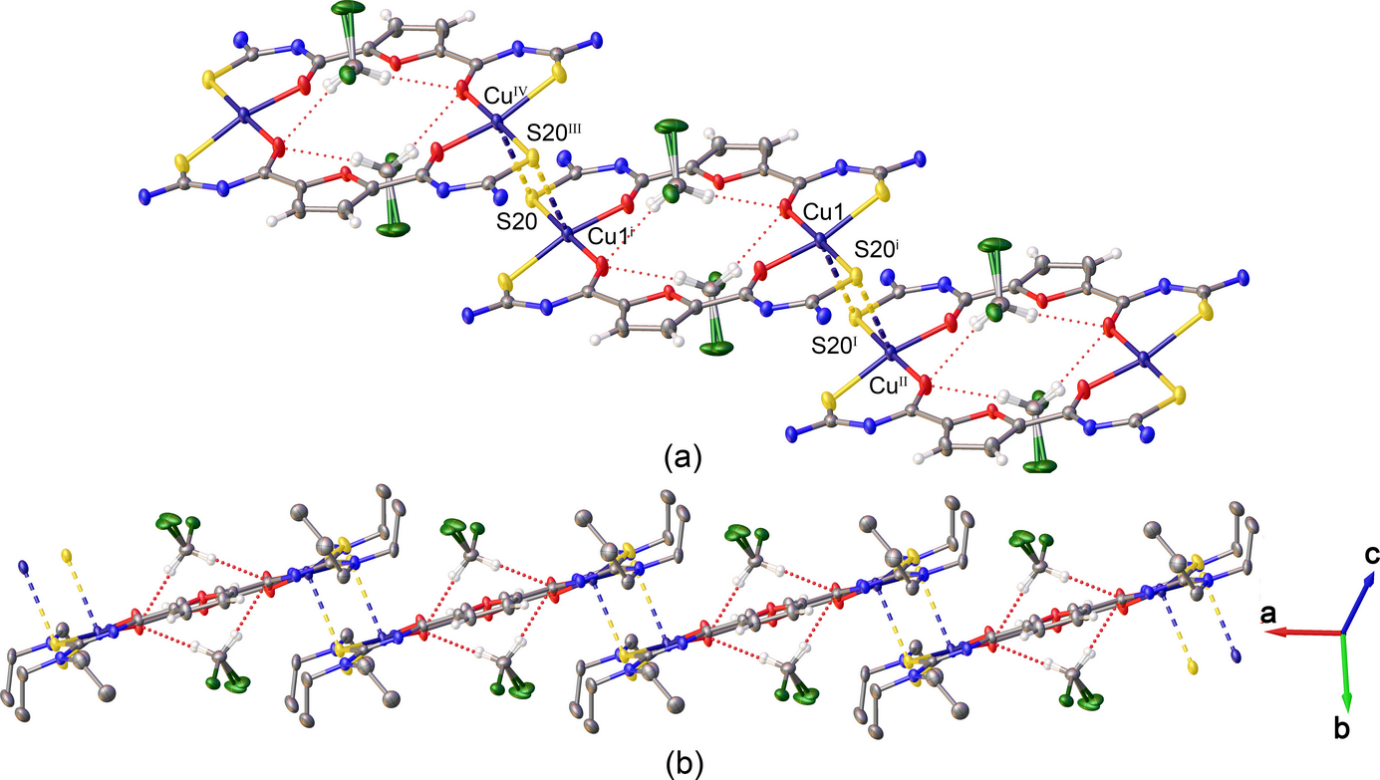
(*a*) Mol­ecular packing of [Cu_2_(*L*)_2_]·2CH_2_Cl_2_ units by coordinative Cu⋯S inter­actions (dashed lines). Symmetry codes: (i) −*x* + 1, −*y* + 1, −*z* + 1; (I)*x* + 1, *y*, *z*; (II) −*x* + 2, −*y* + 1, −*z* + 1; (III) −*x*, −*y* + 1, −*z* + 1; (IV) *x* − 1, *y*, *z*. Ethyl groups are omitted for clarity. (*b*) Polymeric chains along the *a-*axis direction. Hydrogen atoms of ethyl groups are omitted for clarity.

Each [Cu_2_(*L*)_2_]·2CH_2_Cl_2_ unit inter­acts with two adjacent ones by long bonding inter­actions between the Cu^II^ ions and S20 atoms of adjacent blocks (Fig. 2 ▸*a*). These bonds, with a distance of 2.9884 (6) Å, are considerably longer than the coordinative Cu—S bonds within the [Cu_2_(*L*)_2_] unit. Such inter­actions between the units results in polymeric chains along the *a-*axis direction (Fig. 2 ▸*b*).

**Figure 3:**
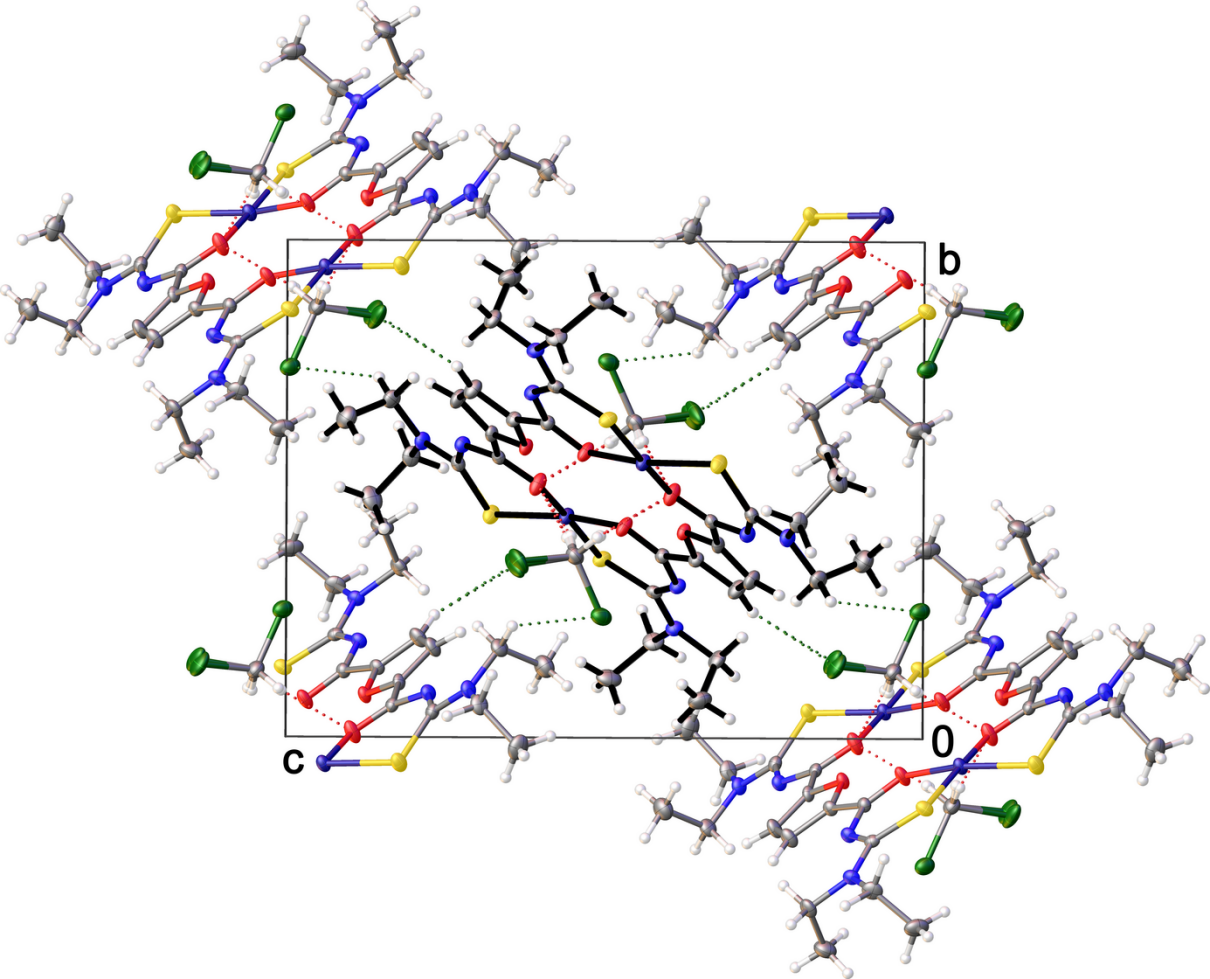
Crystal packing of the title compound shown in projection down the *a-*axis illustrating the aggregation of chains by C—H⋯Cl hydrogen bonding (green dotted lines). The central units are highlighted for clarity.

Furthermore, inter­molecular hydrogen bonds (Table 1 ▸) involving the solvent mol­ecules and the C—H bonds of the furan rings and ethyl groups are responsible for aggregation of the polymeric chains (Fig. 3 ▸).

## 4. Database survey

The crystal structures of neither the ligand nor its metal complexes are found in the Cambridge Structure Database (CSD version 5.45, update of June 2024; Groom *et al.*, 2016 ▸). A search of the CSD for dinuclear copper(II) complexes derived from aroylbis(thio­ureas) reveals only five hits involving isophthaloyl derivatives: DIZTEM and DIZTEM1 (Rodenstein *et al.*, 2008 ▸), BEWKAR (Schwade *et al.*, 2013 ▸), DOMNIE (Selvakumaran *et al.*, 2014 ▸) and YUFNUL (Teixeira *et al.*, 2020 ▸). Across the series of metrics for these structures, all values regarding the coordination of copper(II) ions and aroyl­thio­urea moieties are in accordance with those reported herein.

## 5. Synthesis and crystallization

**H_2_L** (38.5 mg, 0.1 mmol) was added into a solution of CuCl_2_·2H_2_O (17.1 mg, 0.1 mmol) in 1 mL of MeOH. The reaction mixture was stirred at 313 K for 30 min before adding the supporting base Et_3_N (0.03 mL, 0.2 mmol). A brown precipitate deposited immediately. After stirring for additional 1 h at 313 K, the product was filtered off, washed with MeOH, and dried under reduced pressure. Single crystals suitable for X-ray analysis were obtained by slow evaporation of a solution of the complex in a mixture of CH_2_Cl_2_ and MeOH. Under ambient conditions, the crystals slowly turned to powder due to the evaporation of the co-crystalized solvent.

IR (KBr, cm^−1^): 2974 (*w*), 2931 (*w*), 1536 (*s*), 1492 (*s*), 1455 (*m*), 1399 (*s*), 1373 (*s*), 1348 (*s*), 1304 (*s*), 1262 (*s*), 1219 (*m*), 1148 (*m*), 1111 (*m*), 1074 (*m*), 1008 (*s*), 9722 (*m*), 880 (*s*), 813 (*s*), 767 (*s*), 665 (*m*), 6155 (*w*), 548 (*w*), 455 (*m*).

+ESI MS (*m*/*z*): 893.19 (calculated 893.09), 50% [Cu_2_(*L*)_2_ + H]^+^; 931.24 (calculated 931.05), 100% [Cu_2_(*L*)_2_ + K]^+^.

## 6. Refinement

Crystal data, data collection and structure refinement details are summarized in Table 2 ▸. The H atoms were placed at calculated positions and refined in riding mode, with C—H distances of 0.95 Å (aromatic), 0.99 Å (CH_2_) and 0.98 Å (CH_3_), and isotropic displacement parameters equal to 1.2*U*_eq_ of the parent atoms (1.5*U*_eq_ for CH_3_).

## Supplementary Material

Crystal structure: contains datablock(s) I. DOI: 10.1107/S2056989024010703/oo2007sup1.cif

Structure factors: contains datablock(s) I. DOI: 10.1107/S2056989024010703/oo2007Isup2.hkl

CCDC reference: 2400458

Additional supporting information:  crystallographic information; 3D view; checkCIF report

## Figures and Tables

**Table 1:** Hydrogen-bond geometry (Å, °)

*D*—H⋯*A*	*D*—H	H⋯*A*	*D*⋯*A*	*D*—H⋯*A*
C30—H30*A*⋯O10	0.99	2.41	3.388 (3)	171
C30—H30*B*⋯O10^i^	0.99	2.45	3.428 (3)	171
C14—H14*B*⋯Cl1^ii^	0.99	2.83	3.627 (2)	138
C4—H4⋯Cl2*A*^iii^	0.95	2.68	3.506 (8)	145
C4—H4⋯Cl2*B*^iii^	0.95	2.60	3.446 (12)	149

Symmetry codes: (i) 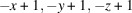; (ii) 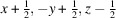; (iii) 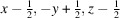.

**Table 2:** Experimental details

Crystal data	
Chemical formula	[Cu_2_(C_16_H_22_N_4_O_3_S_2_)_2_]·2CH_2_Cl_2_
*M*_r_	1061.92
Crystal system, space group	Monoclinic, *P*2_1_/*n*
Temperature (K)	140
*a*, *b*, *c* (Å)	10.2290 (9), 13.0681 (10), 16.9601 (15)
β (°)	98.377 (3)
*V* (Å^3^)	2242.9 (3)
*Z*	2
Radiation type	Mo *K*α
μ (mm^−1^)	1.42
Crystal size (mm)	0.14 × 0.08 × 0.05

Data collection	
Diffractometer	Bruker APEXII CCD
Absorption correction	Multi-scan (*SADABS*; Krause *et al.*, 2015 ▸)
*T*_min_, *T*_max_	0.686, 0.746
No. of measured, independent and observed [*I* > 2σ(*I*)] reflections	29179, 5809, 4232
*R*_int_	0.062
(sin θ/λ)_max_ (Å^−1^)	0.677

Refinement	
*R*[*F*^2^ > 2σ(*F*^2^)], *wR*(*F*^2^), *S*	0.038, 0.076, 1.03
No. of reflections	5809
No. of parameters	276
H-atom treatment	H-atom parameters constrained
Δρ_max_, Δρ_min_ (e Å^−3^)	0.50, −0.45

Computer programs: *APEX2* ans *SAINT* (Bruker, 2014 ▸), *SHELXT2014/5* (Sheldrick, 2015*a* ▸), *SHELXL2018/3* (Sheldrick, 2015*b* ▸) and *OLEX2* (Dolomanov *et al.*, 2009 ▸).

## supplementary crystallographic information

### Bis[µ-3,3,3',3'-tetraethyl-1,1'-(furan-2,5-dicarbonyl)bis(thioureato)]dicopper(II) dichloromethane disolvate Crystal data

**Table:**

[Cu_2_(C_16_H_22_N_4_O_3_S_2_)_2_]·2CH_2_Cl_2_	*F*(000) = 1092
*M**_r_* = 1061.92	*D*_x_ = 1.572 Mg m^−^^3^
Monoclinic, *P*2_1_/*n*	Mo *K*α radiation, λ = 0.71073 Å
*a* = 10.2290 (9) Å	Cell parameters from 8429 reflections
*b* = 13.0681 (10) Å	θ = 2.9–28.6°
*c* = 16.9601 (15) Å	µ = 1.42 mm^−^^1^
β = 98.377 (3)°	*T* = 140 K
*V* = 2242.9 (3) Å^3^	Plate, red
*Z* = 2	0.14 × 0.08 × 0.05 mm

### Bis[µ-3,3,3',3'-tetraethyl-1,1'-(furan-2,5-dicarbonyl)bis(thioureato)]dicopper(II) dichloromethane disolvate Data collection

**Table:**

Bruker APEXII CCD diffractometer	4232 reflections with *I* > 2σ(*I*)
φ and ω scans	*R*_int_ = 0.062
Absorption correction: multi-scan (SADABS; Krause *et al.*, 2015)	θ_max_ = 28.8°, θ_min_ = 2.9°
*T*_min_ = 0.686, *T*_max_ = 0.746	*h* = −13→12
29179 measured reflections	*k* = −16→17
5809 independent reflections	*l* = −22→22

### Bis[µ-3,3,3',3'-tetraethyl-1,1'-(furan-2,5-dicarbonyl)bis(thioureato)]dicopper(II) dichloromethane disolvate Refinement

**Table:**

Refinement on *F*^2^	Primary atom site location: dual
Least-squares matrix: full	Hydrogen site location: inferred from neighbouring sites
*R*[*F*^2^ > 2σ(*F*^2^)] = 0.038	H-atom parameters constrained
*wR*(*F*^2^) = 0.076	*w* = 1/[σ^2^(*F*_o_^2^) + (0.0292*P*)^2^ + 0.9669*P*] where *P* = (*F*_o_^2^ + 2*F*_c_^2^)/3
*S* = 1.03	(Δ/σ)_max_ = 0.001
5809 reflections	Δρ_max_ = 0.50 e Å^−^^3^
276 parameters	Δρ_min_ = −0.44 e Å^−^^3^
0 restraints	

### Bis[µ-3,3,3',3'-tetraethyl-1,1'-(furan-2,5-dicarbonyl)bis(thioureato)]dicopper(II) dichloromethane disolvate Special details

**Table:**

Geometry. All esds (except the esd in the dihedral angle between two l.s. planes) are estimated using the full covariance matrix. The cell esds are taken into account individually in the estimation of esds in distances, angles and torsion angles; correlations between esds in cell parameters are only used when they are defined by crystal symmetry. An approximate (isotropic) treatment of cell esds is used for estimating esds involving l.s. planes.

### Bis[µ-3,3,3',3'-tetraethyl-1,1'-(furan-2,5-dicarbonyl)bis(thioureato)]dicopper(II) dichloromethane disolvate Fractional atomic coordinates and isotropic or equivalent isotropic displacement parameters (Å^2^)

**Table:**

	*x*	*y*	*z*	*U*_iso_*/*U*_eq_	Occ. (<1)
Cu1	0.84517 (2)	0.55425 (2)	0.43977 (2)	0.01612 (8)	
S20	−0.02763 (5)	0.36002 (4)	0.50470 (3)	0.01813 (12)	
S10	0.92190 (5)	0.55137 (4)	0.32124 (3)	0.02175 (13)	
Cl1	0.51955 (6)	0.24405 (4)	0.50494 (4)	0.02871 (14)	
Cl2A	0.6845 (12)	0.3546 (8)	0.6351 (5)	0.0389 (13)	0.62 (6)
O1	0.43749 (13)	0.41530 (10)	0.37590 (8)	0.0148 (3)	
O20	0.24820 (14)	0.42489 (11)	0.46961 (9)	0.0201 (3)	
O10	0.67999 (14)	0.49014 (12)	0.39462 (9)	0.0234 (4)	
N20	0.12097 (16)	0.30601 (13)	0.38549 (10)	0.0161 (4)	
N10	0.72045 (17)	0.41079 (13)	0.27587 (11)	0.0171 (4)	
N21	−0.06963 (17)	0.22096 (13)	0.39037 (11)	0.0174 (4)	
N11	0.90007 (17)	0.40368 (13)	0.21277 (11)	0.0181 (4)	
C20	0.2228 (2)	0.36677 (15)	0.40972 (12)	0.0155 (4)	
C21	0.01399 (19)	0.29379 (15)	0.42245 (12)	0.0149 (4)	
C10	0.6509 (2)	0.43597 (15)	0.33275 (12)	0.0146 (4)	
C5	0.3243 (2)	0.35962 (15)	0.35652 (13)	0.0156 (4)	
C2	0.5189 (2)	0.38896 (16)	0.32183 (12)	0.0150 (4)	
C11	0.8417 (2)	0.44890 (16)	0.26910 (12)	0.0165 (4)	
C4	0.3331 (2)	0.29970 (17)	0.29214 (14)	0.0226 (5)	
H4	0.267641	0.253875	0.267244	0.027*	
C12	1.0320 (2)	0.43485 (17)	0.19544 (14)	0.0221 (5)	
H12A	1.076331	0.374578	0.175901	0.026*	
H12B	1.085855	0.457912	0.245532	0.026*	
C14	0.8325 (2)	0.31971 (17)	0.16420 (13)	0.0229 (5)	
H14A	0.784256	0.276870	0.198538	0.027*	
H14B	0.899900	0.276039	0.144445	0.027*	
C3	0.4587 (2)	0.31868 (17)	0.26922 (13)	0.0218 (5)	
H3	0.493828	0.288489	0.225885	0.026*	
C22	−0.1906 (2)	0.19430 (17)	0.42337 (14)	0.0237 (5)	
H22A	−0.255015	0.163290	0.380844	0.028*	
H22B	−0.230516	0.257583	0.441381	0.028*	
C30	0.5687 (2)	0.36547 (17)	0.54694 (14)	0.0244 (5)	
H30A	0.608215	0.406292	0.507265	0.029*	0.62 (6)
H30B	0.489631	0.402474	0.559208	0.029*	0.62 (6)
H30C	0.620516	0.402930	0.511272	0.029*	0.38 (6)
H30D	0.489701	0.406736	0.553233	0.029*	0.38 (6)
C24	−0.0367 (2)	0.15816 (17)	0.32306 (14)	0.0233 (5)	
H24A	−0.002603	0.203498	0.283944	0.028*	
H24B	−0.118227	0.125169	0.296061	0.028*	
C23	−0.1646 (3)	0.12006 (18)	0.49296 (16)	0.0324 (6)	
H23A	−0.122811	0.057995	0.475893	0.049*	
H23B	−0.248397	0.102117	0.511037	0.049*	
H23C	−0.105823	0.152244	0.536775	0.049*	
C15	0.7365 (3)	0.3571 (2)	0.09391 (15)	0.0333 (6)	
H15A	0.784371	0.396006	0.057839	0.050*	
H15B	0.670058	0.401186	0.112873	0.050*	
H15C	0.692659	0.298337	0.065448	0.050*	
C13	1.0272 (3)	0.5201 (2)	0.13398 (16)	0.0336 (6)	
H13A	0.975577	0.497390	0.083784	0.050*	
H13B	1.117258	0.536891	0.125049	0.050*	
H13C	0.985739	0.580787	0.153507	0.050*	
C25	0.0658 (3)	0.07585 (18)	0.34959 (17)	0.0336 (6)	
H25A	0.087206	0.038988	0.302800	0.050*	
H25B	0.030115	0.027765	0.385352	0.050*	
H25C	0.145957	0.107842	0.377676	0.050*	
Cl2B	0.6656 (19)	0.3460 (10)	0.6407 (9)	0.044 (2)	0.38 (6)

### Bis[µ-3,3,3',3'-tetraethyl-1,1'-(furan-2,5-dicarbonyl)bis(thioureato)]dicopper(II) dichloromethane disolvate Atomic displacement parameters (Å^2^)

**Table:**

	*U*^11^	*U*^22^	*U*^33^	*U*^12^	*U*^13^	*U*^23^
Cu1	0.01290 (13)	0.02103 (14)	0.01530 (14)	−0.00403 (10)	0.00497 (10)	−0.00410 (11)
S20	0.0141 (3)	0.0184 (3)	0.0231 (3)	−0.0034 (2)	0.0069 (2)	−0.0044 (2)
S10	0.0214 (3)	0.0267 (3)	0.0191 (3)	−0.0083 (2)	0.0096 (2)	−0.0042 (2)
Cl1	0.0350 (3)	0.0237 (3)	0.0301 (3)	0.0012 (2)	0.0136 (3)	0.0036 (2)
Cl2A	0.041 (2)	0.045 (2)	0.0275 (12)	0.006 (2)	−0.0045 (13)	0.0173 (14)
O1	0.0119 (7)	0.0207 (7)	0.0127 (8)	−0.0033 (6)	0.0046 (6)	−0.0045 (6)
O20	0.0158 (7)	0.0274 (8)	0.0184 (8)	−0.0086 (6)	0.0074 (6)	−0.0106 (6)
O10	0.0135 (8)	0.0379 (9)	0.0199 (9)	−0.0081 (7)	0.0067 (6)	−0.0118 (7)
N20	0.0125 (9)	0.0177 (9)	0.0181 (10)	−0.0029 (7)	0.0023 (7)	−0.0029 (7)
N10	0.0156 (9)	0.0200 (9)	0.0168 (10)	−0.0010 (7)	0.0063 (8)	−0.0021 (7)
N21	0.0133 (9)	0.0172 (9)	0.0217 (10)	−0.0030 (7)	0.0022 (7)	−0.0021 (7)
N11	0.0150 (9)	0.0240 (9)	0.0166 (10)	0.0002 (7)	0.0068 (8)	−0.0021 (8)
C20	0.0142 (10)	0.0174 (10)	0.0146 (11)	−0.0015 (8)	0.0014 (9)	−0.0008 (8)
C21	0.0124 (10)	0.0134 (10)	0.0184 (11)	0.0005 (8)	0.0010 (9)	0.0019 (8)
C10	0.0144 (10)	0.0163 (10)	0.0129 (11)	0.0013 (8)	0.0018 (8)	0.0019 (8)
C5	0.0124 (10)	0.0177 (10)	0.0165 (11)	−0.0032 (8)	0.0010 (8)	−0.0025 (8)
C2	0.0141 (10)	0.0204 (11)	0.0114 (11)	0.0029 (8)	0.0046 (8)	−0.0012 (8)
C11	0.0156 (10)	0.0199 (10)	0.0141 (11)	0.0012 (9)	0.0022 (8)	0.0026 (9)
C4	0.0173 (11)	0.0275 (12)	0.0232 (13)	−0.0066 (9)	0.0036 (10)	−0.0097 (10)
C12	0.0134 (11)	0.0320 (12)	0.0224 (12)	0.0028 (9)	0.0078 (9)	−0.0027 (10)
C14	0.0230 (12)	0.0251 (12)	0.0222 (13)	0.0030 (10)	0.0084 (10)	−0.0045 (9)
C3	0.0201 (11)	0.0279 (12)	0.0189 (12)	−0.0038 (9)	0.0079 (10)	−0.0092 (9)
C22	0.0142 (11)	0.0241 (12)	0.0331 (14)	−0.0086 (9)	0.0044 (10)	−0.0022 (10)
C30	0.0273 (13)	0.0233 (12)	0.0222 (13)	0.0032 (10)	0.0028 (10)	0.0042 (9)
C24	0.0221 (12)	0.0257 (12)	0.0215 (12)	−0.0086 (10)	0.0013 (10)	−0.0080 (9)
C23	0.0313 (14)	0.0234 (13)	0.0458 (17)	−0.0043 (11)	0.0165 (12)	0.0046 (11)
C15	0.0300 (14)	0.0382 (15)	0.0303 (15)	−0.0026 (11)	−0.0004 (11)	−0.0064 (11)
C13	0.0251 (13)	0.0474 (16)	0.0303 (15)	−0.0067 (12)	0.0108 (11)	0.0089 (12)
C25	0.0389 (15)	0.0242 (13)	0.0392 (16)	0.0012 (11)	0.0106 (13)	−0.0070 (11)
Cl2B	0.056 (4)	0.043 (2)	0.027 (3)	−0.021 (3)	−0.016 (3)	0.0175 (18)

### Bis[µ-3,3,3',3'-tetraethyl-1,1'-(furan-2,5-dicarbonyl)bis(thioureato)]dicopper(II) dichloromethane disolvate Geometric parameters (Å, º)

**Table:**

Cu1—S20^i^	2.2612 (6)	C12—H12B	0.9900
Cu1—S10	2.2624 (6)	C12—C13	1.521 (3)
Cu1—O20^i^	1.9431 (14)	C14—H14A	0.9900
Cu1—O10	1.9406 (15)	C14—H14B	0.9900
S20—C21	1.746 (2)	C14—C15	1.511 (3)
S10—C11	1.743 (2)	C3—H3	0.9500
Cl1—C30	1.781 (2)	C22—H22A	0.9900
Cl2A—C30	1.773 (8)	C22—H22B	0.9900
O1—C5	1.366 (2)	C22—C23	1.521 (3)
O1—C2	1.369 (2)	C30—H30A	0.9900
O20—C20	1.265 (2)	C30—H30B	0.9900
O10—C10	1.265 (2)	C30—H30C	0.9900
N20—C20	1.326 (3)	C30—H30D	0.9900
N20—C21	1.347 (3)	C30—Cl2B	1.766 (13)
N10—C10	1.321 (3)	C24—H24A	0.9900
N10—C11	1.357 (3)	C24—H24B	0.9900
N21—C21	1.341 (3)	C24—C25	1.523 (3)
N21—C22	1.472 (3)	C23—H23A	0.9800
N21—C24	1.484 (3)	C23—H23B	0.9800
N11—C11	1.336 (3)	C23—H23C	0.9800
N11—C12	1.479 (3)	C15—H15A	0.9800
N11—C14	1.481 (3)	C15—H15B	0.9800
C20—C5	1.475 (3)	C15—H15C	0.9800
C10—C2	1.471 (3)	C13—H13A	0.9800
C5—C4	1.357 (3)	C13—H13B	0.9800
C2—C3	1.363 (3)	C13—H13C	0.9800
C4—H4	0.9500	C25—H25A	0.9800
C4—C3	1.417 (3)	C25—H25B	0.9800
C12—H12A	0.9900	C25—H25C	0.9800
			
S20^i^—Cu1—S10	90.35 (2)	C2—C3—C4	106.24 (19)
O20^i^—Cu1—S20^i^	94.14 (4)	C2—C3—H3	126.9
O20^i^—Cu1—S10	168.29 (5)	C4—C3—H3	126.9
O10—Cu1—S20^i^	175.25 (5)	N21—C22—H22A	109.1
O10—Cu1—S10	92.12 (5)	N21—C22—H22B	109.1
O10—Cu1—O20^i^	82.68 (6)	N21—C22—C23	112.59 (19)
C21—S20—Cu1^i^	107.20 (7)	H22A—C22—H22B	107.8
C11—S10—Cu1	105.37 (7)	C23—C22—H22A	109.1
C5—O1—C2	106.35 (15)	C23—C22—H22B	109.1
C20—O20—Cu1^i^	130.70 (13)	Cl1—C30—H30A	109.1
C10—O10—Cu1	130.91 (13)	Cl1—C30—H30B	109.1
C20—N20—C21	125.52 (18)	Cl1—C30—H30C	110.0
C10—N10—C11	124.50 (18)	Cl1—C30—H30D	110.0
C21—N21—C22	122.38 (18)	Cl2A—C30—Cl1	112.4 (4)
C21—N21—C24	120.11 (17)	Cl2A—C30—H30A	109.1
C22—N21—C24	117.34 (17)	Cl2A—C30—H30B	109.1
C11—N11—C12	122.41 (18)	H30A—C30—H30B	107.9
C11—N11—C14	120.30 (17)	H30C—C30—H30D	108.3
C12—N11—C14	117.29 (17)	Cl2B—C30—Cl1	108.7 (5)
O20—C20—N20	131.98 (19)	Cl2B—C30—H30C	110.0
O20—C20—C5	116.66 (17)	Cl2B—C30—H30D	110.0
N20—C20—C5	111.33 (18)	N21—C24—H24A	109.0
N20—C21—S20	128.46 (16)	N21—C24—H24B	109.0
N21—C21—S20	117.42 (15)	N21—C24—C25	112.76 (19)
N21—C21—N20	114.12 (18)	H24A—C24—H24B	107.8
O10—C10—N10	131.22 (19)	C25—C24—H24A	109.0
O10—C10—C2	116.03 (17)	C25—C24—H24B	109.0
N10—C10—C2	112.72 (18)	C22—C23—H23A	109.5
O1—C5—C20	117.82 (17)	C22—C23—H23B	109.5
C4—C5—O1	110.36 (17)	C22—C23—H23C	109.5
C4—C5—C20	131.58 (19)	H23A—C23—H23B	109.5
O1—C2—C10	116.58 (17)	H23A—C23—H23C	109.5
C3—C2—O1	110.28 (18)	H23B—C23—H23C	109.5
C3—C2—C10	133.07 (19)	C14—C15—H15A	109.5
N10—C11—S10	127.42 (16)	C14—C15—H15B	109.5
N11—C11—S10	118.44 (15)	C14—C15—H15C	109.5
N11—C11—N10	114.05 (18)	H15A—C15—H15B	109.5
C5—C4—H4	126.6	H15A—C15—H15C	109.5
C5—C4—C3	106.76 (19)	H15B—C15—H15C	109.5
C3—C4—H4	126.6	C12—C13—H13A	109.5
N11—C12—H12A	108.9	C12—C13—H13B	109.5
N11—C12—H12B	108.9	C12—C13—H13C	109.5
N11—C12—C13	113.50 (19)	H13A—C13—H13B	109.5
H12A—C12—H12B	107.7	H13A—C13—H13C	109.5
C13—C12—H12A	108.9	H13B—C13—H13C	109.5
C13—C12—H12B	108.9	C24—C25—H25A	109.5
N11—C14—H14A	108.9	C24—C25—H25B	109.5
N11—C14—H14B	108.9	C24—C25—H25C	109.5
N11—C14—C15	113.34 (19)	H25A—C25—H25B	109.5
H14A—C14—H14B	107.7	H25A—C25—H25C	109.5
C15—C14—H14A	108.9	H25B—C25—H25C	109.5
C15—C14—H14B	108.9		
			
Cu1^i^—S20—C21—N20	−14.0 (2)	C10—N10—C11—S10	−11.0 (3)
Cu1^i^—S20—C21—N21	166.21 (14)	C10—N10—C11—N11	172.43 (19)
Cu1—S10—C11—N10	29.0 (2)	C10—C2—C3—C4	176.6 (2)
Cu1—S10—C11—N11	−154.56 (15)	C5—O1—C2—C10	−177.26 (17)
Cu1^i^—O20—C20—N20	5.2 (4)	C5—O1—C2—C3	0.3 (2)
Cu1^i^—O20—C20—C5	−172.26 (14)	C5—C4—C3—C2	0.4 (3)
Cu1—O10—C10—N10	−5.1 (3)	C2—O1—C5—C20	175.01 (18)
Cu1—O10—C10—C2	172.61 (14)	C2—O1—C5—C4	0.0 (2)
O1—C5—C4—C3	−0.2 (3)	C11—N10—C10—O10	−6.7 (4)
O1—C2—C3—C4	−0.4 (3)	C11—N10—C10—C2	175.57 (19)
O20—C20—C5—O1	0.3 (3)	C11—N11—C12—C13	−88.5 (3)
O20—C20—C5—C4	174.1 (2)	C11—N11—C14—C15	83.8 (2)
O10—C10—C2—O1	5.2 (3)	C12—N11—C11—S10	2.4 (3)
O10—C10—C2—C3	−171.6 (2)	C12—N11—C11—N10	179.34 (18)
N20—C20—C5—O1	−177.58 (17)	C12—N11—C14—C15	−95.6 (2)
N20—C20—C5—C4	−3.8 (3)	C14—N11—C11—S10	−176.94 (15)
N10—C10—C2—O1	−176.69 (17)	C14—N11—C11—N10	0.0 (3)
N10—C10—C2—C3	6.5 (3)	C14—N11—C12—C13	90.9 (2)
C20—N20—C21—S20	6.1 (3)	C22—N21—C21—S20	−1.7 (3)
C20—N20—C21—N21	−174.10 (19)	C22—N21—C21—N20	178.48 (18)
C20—C5—C4—C3	−174.3 (2)	C22—N21—C24—C25	−98.7 (2)
C21—N20—C20—O20	0.9 (4)	C24—N21—C21—S20	−176.80 (15)
C21—N20—C20—C5	178.46 (19)	C24—N21—C21—N20	3.4 (3)
C21—N21—C22—C23	−82.9 (2)	C24—N21—C22—C23	92.4 (2)
C21—N21—C24—C25	76.7 (2)		

Symmetry code: (i) −*x*+1, −*y*+1, −*z*+1.

### Bis[µ-3,3,3',3'-tetraethyl-1,1'-(furan-2,5-dicarbonyl)bis(thioureato)]dicopper(II) dichloromethane disolvate Hydrogen-bond geometry (Å, º)

**Table:**

*D*—H···*A*	*D*—H	H···*A*	*D*···*A*	*D*—H···*A*
C30—H30*A*···O10	0.99	2.41	3.388 (3)	171
C30—H30*B*···O10^i^	0.99	2.45	3.428 (3)	171
C14—H14*B*···Cl1^ii^	0.99	2.83	3.627 (2)	138
C4—H4···Cl2*A*^iii^	0.95	2.68	3.506 (8)	145
C4—H4···Cl2*B*^iii^	0.95	2.60	3.446 (12)	149

Symmetry codes: (i) −*x*+1, −*y*+1, −*z*+1; (ii) *x*+1/2, −*y*+1/2, *z*−1/2; (iii) *x*−1/2, −*y*+1/2, *z*−1/2.
